# Energy Metabolism Enhance Perylenequinone Biosynthesis in *Shiraia* sp. Slf14 through Promoting Mitochondrial ROS Accumulation

**DOI:** 10.3390/ijms251810113

**Published:** 2024-09-20

**Authors:** Xueyi Wu, Xuan Meng, Yiwen Xiao, Huilin Yang, Zhibin Zhang, Du Zhu

**Affiliations:** 1 College of Life Sciences, Jiangxi Normal University, Nanchang 330022, China; wuxueyi0607@163.com (X.W.); mengx9807@163.com (X.M.); yanghl@jxnu.edu.cn (H.Y.); 2 Key Laboratory of Natural Microbial Medicine Research of Jiangxi Province, Jiangxi Science and Technology Normal University, Nanchang 330013, China; xyw1152858687@163.com; 3 Key Laboratory of Microbial Resources and Metabolism of Nanchang City, Jiangxi Science and Technology Normal University, Nanchang 330013, China

**Keywords:** *Shiraia* sp. Slf14, perylenequinones, reactive oxygen species, energy metabolism

## Abstract

Perylenequinones (PQs) are important natural compounds that have been extensively utilized in recent years as agents for antimicrobial, anticancer, and antiviral photodynamic therapies. In this study, we investigated the molecular mechanisms regulating PQ biosynthesis by comparing *Shiraia* sp. Slf14 with its low PQ titer mutant, Slf14(w). The results indicated that the strain Slf14 exhibited a higher PQ yield, a more vigorous energy metabolism, and a more pronounced oxidation state compared to Slf14(w). Transcriptome analysis consistently revealed that the differences in gene expression between Slf14 and Slf14(w) are primarily associated with genes involved in redox processes and energy metabolism. Additionally, reactive oxygen species (ROS) were shown to play a crucial role in promoting PQ synthesis, as evidenced by the application of ROS-related inhibitors and promoters. Further results demonstrated that mitochondria are significant sources of ROS, which effectively regulate PQ biosynthesis in *Shiraia* sp. Slf14. In summary, this research revealed a noteworthy finding: the higher energy metabolism of the strain Slf14 is associated with increased intracellular ROS accumulation, which in turn triggers the activation and expression of gene clusters responsible for PQ synthesis.

## 1. Introduction

Perylenequinones (PQs), characterized by a dihydroxyperylene configuration as their core structure, have been integral components of traditional Chinese herbal treatments for ailments, such as gastric diseases and rheumatoid arthritis [1,2]. The distinctive central five-membered ring structure of PQs confers specific and noteworthy biological activities [3]. Notably, PQs exhibit photosensitizing activity in the UV-visible absorption spectral region, making them highly promising as photodynamic therapeutic agents for antimicrobial, antiviral, and antitumor applications [4,5,6].

Currently, PQs are primarily extracted from the stromata of the bamboo pathogenic fungi *Hypocrella bambusae* and *Shiraia bambusicola*. However, the scarcity of wild fruiting bodies poses significant challenges to PQ production [7]. Consequently, submerged fermentation (SmF) utilizing the mycelium of *Shiraia* spp. has emerged as a promising alternative for PQ production [1,8]. Thus far, most prior efforts have focused on enhancing the PQ yield in the SmF of *Shiraia* spp. through various strategies, including medium optimization [7,8,9], mutagenesis breeding [10], and the addition of inducers [11,12]. Additionally, Su et al. [13] synthesized a series of non-natural PQs using a biosynthetic approach. Nevertheless, the current PQ yield remains insufficient to meet market demands [1,10]. Therefore, there is a compelling need to optimize the SmF process and gain a deeper understanding of PQ biosynthesis mechanisms to enhance PQ production [1].

Reactive oxygen species (ROS) refers to a group of highly reactive, oxygen-containing substances with strong oxidant properties, generated as by-products during aerobic metabolism or accumulated in response to stress [14,15]. Excessive levels of ROS can lead to irreversible damage to intracellular components [16,17]. Oxidative stress occurs when ROS production escalates or when ROS scavenging mechanisms are disrupted [18]. Notably, lower concentrations of ROS can act as signaling molecules in cascades that ultimately regulate gene expression and facilitate adaptation. Increasingly, ROS are recognized as versatile intracellular signaling mediators that play a pivotal role in regulating various physiological and biological responses in filamentous fungi. For example, in *Penicillium* spp., changes in intracellular ROS levels indirectly influence the production of penicillin and cephalosporin [19]. The precise control of the ROS concentration and redox potential emerges as one of the most critical factors in fungal cell signaling [20,21]. In a broader context, the production of secondary metabolites is not a continuous process; rather, it is often triggered by the organism’s development or specific events [22]. Numerous studies have established a connection between oxidative stress and secondary metabolism [23,24]. Elevated intracellular ROS levels, for instance, have been shown to significantly enhance the content of ganoderic acid (GA) in *Ganoderma lucidum* [25]. Furthermore, oxidative stress can exacerbate aflatoxin production in *Aspergillus flavus* [26]. The application of chemicals to block signaling pathways or induce oxidative stress has proven effective in inhibiting mycotoxin production [27], thereby underscoring the link between oxidative stress and the regulation of secondary metabolism in filamentous fungi.

Mitochondria play a central role in ATP generation through oxidative phosphorylation, thereby positioning them at the core of energy metabolism [28]. In addition to facilitating the transport of nutrients for ATP production, mitochondria also produce intermediates for biosynthesis and generate ROS. These ROS can function as additional messengers that orchestrate signal transduction and metabolic processes [29]. Typically, the flow of electrons into the mitochondrial respiratory chain governs the production of oxygen radicals and other reactive species [30]. Overall, mitochondria serve a dual role as both a source and a target of cellular ROS [31]. Consequently, mitochondrion-associated energy metabolism demonstrates a profound connection with the biosynthesis of secondary products, with metabolic fluxes from primary metabolism being indispensable for the genes responsible for secondary metabolism [32]. 

Our group previously isolated an endophytic fungus, *Shiraia* sp. Slf14, which is capable of producing PQs as mixtures of hypocrellin A (HA) and elsinochrome A (EA), EB, and EC [33,34,35]. By optimizing the culture conditions, a remarkable PQ production of 1566.64 mg/L was achieved in *Shiraia* sp. Slf14 [35]. Subsequently, during the successive cultures of the strain Slf14, a mutant strain, designated Slf14(w), was isolated, exhibiting a low PQ titer [36]. This led to the revelation of substantial disparities in mycelial growth and PQ biosynthesis between Slf14 and Slf14(w) under SmF conditions. Furthermore, notable differences were observed in the ROS levels and antioxidant enzyme systems between these two strains. Additional analyses revealed significant distinctions in the expression of genes associated with energy metabolism and mitochondrial oxidative phosphorylation. Consequently, further investigations into the energy metabolism-related aspects of these two strains were conducted to unravel the intricate relationship between energy metabolism and PQ production. This study contributes to a deeper understanding of the biosynthetic mechanisms underlying PQ while offering potential insights into the regulation of PQ biosynthesis and the interplay between primary and secondary metabolism.

## 2. Results

### 2.1. Growth and PQ Biosynthesis of Strains Slf14 and Slf14(w)

**Figure 1:**
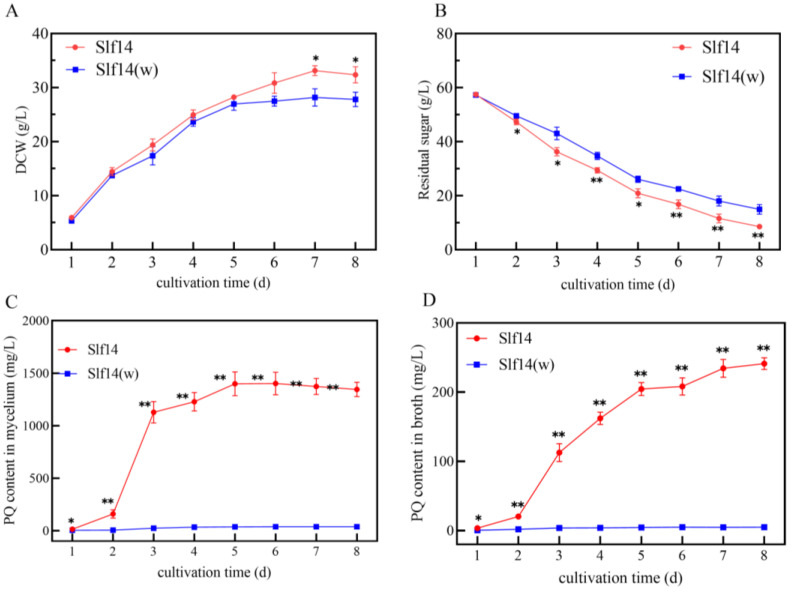
Differences in biomass (**A**), residual sugars (**B**), contents of intracellular PQ (**C**), and extracellular PQ (**D**) during growth of *Shiraia* sp. Slf14 and *Shiraia* sp. Slf14(w). Three biological replicates were performed for each experimental group, * indicates a significant difference *p* < 0.05 and ** indicates a highly significant difference *p* < 0.01.

Initially, strains Slf14 and Slf14(w) exhibited similar growth trends during the first five days, with only minor differences in biomass accumulation. The biomass of the mutant strain Slf14(w) reached a maximum of 28.17 ± 1.61 g/L on day 5, while strain Slf14 continued to increase, achieving a maximum of 33.12 ± 0.90 g/L by day 7 (Figure 1A). In terms of sugar utilization, however, strain Slf14 demonstrated a significantly faster consumption rate beginning on day 2. By the end of fermentation, the residual sugar content in the fermentation broth of strain Slf14 was measured at 8.56 ± 0.92 g/L, compared to 14.96 ± 1.75 g/L for the mutant strain Slf14(w) (Figure 1B). Residual sugars were significantly lower on the second day of fermentation for Slf14 compared to Slf14(w); however, there was no significant difference in biomass between the two at this time. This observation suggests that Slf14 converts a substantial amount of sugar into secondary metabolites rather than utilizing it for maintaining primary metabolism. These results suggest that strain Slf14 exhibits more vigorous energy metabolism than strain Slf14(w). 

In the context of PQ biosynthesis, strain Slf14 initiated substantial PQ production as early as the second day of fermentation. The intracellular and extracellular PQ concentrations for strain Slf14 reached 1128.04 ± 101.82 mg/L and 112.73 ± 12.84 mg/L, respectively, on day 3, which were significantly higher than those of Slf14(w). Subsequently, the PQ yield of strain Slf14 continued to rise, achieving a maximum of 1401.62 ± 108.01 mg/L on day 6. In contrast, the maximum intracellular PQ concentration for strain Slf14(w) was only 37.91 ± 1.46 mg/L (Figure 1C). Furthermore, strain Slf14 consistently released PQ into the fermentation broth, with the extracellular PQ content peaking at 241.14 ± 8.54 mg/L on day 8, while strain Slf14(w) reached only 4.83 ± 0.46 mg/L (Figure 1D). These findings clearly illustrate a substantial disparity in PQ production between strains Slf14 and Slf14(w).

### 2.2. Redox State of Strains Slf14 and Slf14(w)

**Figure 2:**
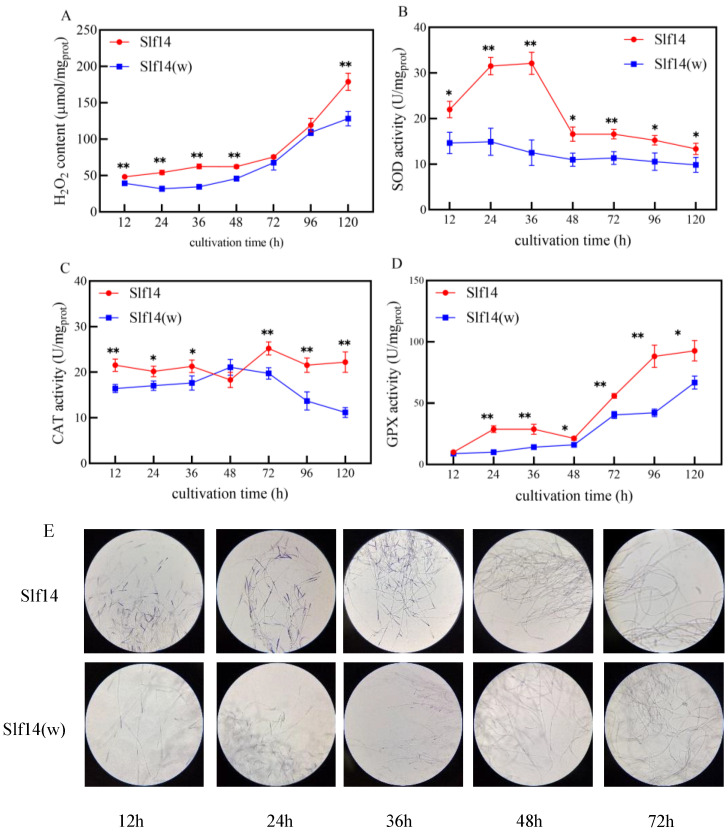
Differences in H_2_O_2_ (**A**), SOD (**B**), CAT (**C**), GPX (**D**), and NBT staining to observe the changes in ROS (**E**) during the growth of *Shiraia* sp. Slf14 and *Shiraia* sp. Slf14(w). The intensity of the staining results serves as a visual indicator of the redox state in both strains during their growth. Notably, a higher concentration of ROS in the mycelium correlates with a bluer hue in the NBT staining. Original magnification: 400×. Three biological replicates were carried out for each experimental group, * indicating a significant difference *p* < 0.05 and ** indicating an extremely significant difference *p* < 0.01.

During the initial growth phase, the levels of hydrogen peroxide (H_2_O_2_) and the activities of antioxidant enzymes were measured in both strains. The results indicated that the H_2_O_2_ content in Slf14 was higher than that in Slf14(w) (Figure 2A). Additionally, the activities of the antioxidant enzymes, including superoxide dismutase (SOD), catalase (CAT), and glutathione peroxidase (GPX), were consistently greater in Slf14 compared to strain Slf14(w) (Figure 2B–D). The intensity of the NBT staining color was directly correlated with the mycelial ROS content, providing a visual representation of changes in ROS levels. Consequently, both strains were stained with NBT during fermentation, revealing a similar redox state (Figure 2E). These findings suggest that Slf14 exhibited elevated intracellular ROS levels during the initial growth phase, with a more pronounced oxidation state compared to Slf14(w).

### 2.3. Transcriptome Analysis Results

Sequencing data statistics for the strains Slf14 and Slf14(w) indicated that, following quality control, the mapping ratios of both clean reads compared to the reference genome exceeded 97%. This finding suggests that the data are suitable for subsequent analyses (Supplementary Materials Table S1). Through bioinformatics analysis, a total of 12,514 genes were compared in this study, of which 1759 were identified as differentially expressed genes (DEGs) (Supplementary Materials Figure S1). To investigate the functional differences between strains Slf14 and Slf14(w), the DEGs obtained from transcriptional analysis were subjected to enrichment analysis. Gene Ontology (GO) enrichment analysis revealed that a significant number of DEGs were associated with “oxidoreductase activity” and “catalytic activity” in molecular function (MF); “oxidation-reduction processes”, “membrane translocation”, and “monobiotic metabolic processes” in biological processes (BPs); and “membrane-intrinsic components” in cellular composition (CC) (Supplementary Materials Figure S2). Furthermore, Kyoto Encyclopedia of Genes and Genomes (KEGG) enrichment analysis demonstrated that a substantial concentration of DEGs was notably enriched in several metabolic pathways, including “glycolysis/gluconeogenesis”, “tyrosine metabolism”, “fatty acid metabolism”, “TCA cycle”, “pyruvate metabolism”, and “carbon metabolism”, as well as pathways related to secondary metabolite biosynthesis and oxidative phosphorylation (Supplementary Materials Figure S3). Collectively, the findings from GO and KEGG analyses suggest that the energy-related metabolism of strain Slf14 is more active than that of strain Slf14(w), which aligns with the rapid sugar consumption of Slf14 (Figure 1B). 

**Figure 3:**
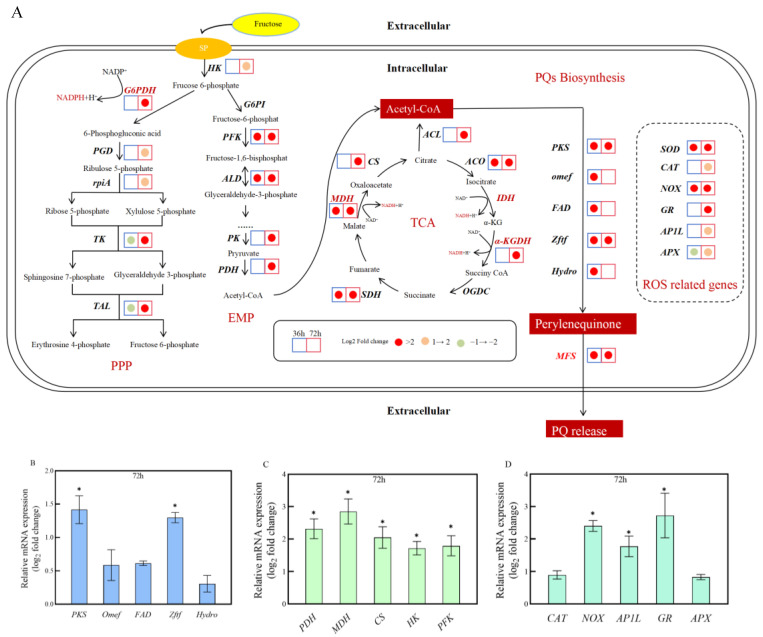
Differences in expression of PQ-related genes, metabolism-related genes, and oxidation-reduction-related genes in *Shiraia* sp. Slf14 and *Shiraia* sp. Slf14(w) at different time periods (**A**). Taking *Shiraia* sp. Slf14(w) at the same period as the control, the picture is based on *Shiraia* sp. Slf14 as the main object. The time periods of 36 h and 72 h were selected. Real-time fluorescence quantitative PCR detection of the relative transcription level of PQ-related genes (**B**), metabolism-related genes (**C**), and oxidation-reduction-related genes (**D**) in *Shiraia* sp. Slf14 and *Shiraia* sp. Slf14(w) at 72 h. * indicates a significant difference compared with the control group (*p* < 0.05).

Based on the results from GO and KEGG analyses, the transcripts related to “secondary metabolites biosynthesis” and “carbon metabolism” were further examined. Compared to the Slf14(w) strain, the genes associated with PQ biosynthesis, including zinc finger transcription (*Zftf*), polyketide synthase(*PKS*), MFS transporter (*MFS*), O-methyltransferase (*Omef*), hydroxylase (*Hydro*), and FAD/FMN-dependent oxidoreductase (*FAD*), were significantly up-regulated between 36 h and 72 h, indicating that Slf14 exhibits enhanced PQ biosynthesis capacity at the transcriptomic level. Additionally, key enzyme genes involved in the glycolysis pathway (EMP), such as hexokinase (*HK*), phosphofructokinase (*PFK*), pyruvate kinase (*PK*), and pyruvate dehydrogenase (*PDH*), demonstrated significant up-regulation in strain Slf14. Furthermore, several genes, such as citrate synthase (*CS*), aconitase (*ACO*), *α*-ketoglutarate dehydrogenase (*α-KGDH*), succinate dehydrgenase (*SDH*), and malate dehydrogenase (*MDH*), associated with the tricarboxylic acid cycle (TCA) also showed up-regulation. Moreover, the genes involved in the pentose phosphate pathway (PPP), which is crucial for providing substantial amounts of NADPH and ribose-5-phosphate necessary for ATP, CoA, DNA, and other essential macromolecules [37,38], were notably up-regulated. The ATP citrate lyase (*ACL*) gene, which catalyzes the cleavage of citric acid to oxaloacetate and acetyl CoA, was significantly up-regulated, suggesting that the fungal cells are capable of generating increased amounts of acetyl CoA for pigment synthesis (Figure 3).

The results of redox-related transcriptome analysis indicated that the expression of several oxidoreductase genes, including *SOD*, *CAT*, *GR*, and *APX*, was up-regulated in strain Slf14 at both 36 h and/or 72 h. Additionally, NAD(P)H oxidase (*NOX*) is recognized as a significant source of ROS [39]. In *Shiraia* sp. S8, the presence of La^3+^ can enhance ROS production by stimulating the NOX complex [40]. Herein, the NOX gene transcript level is also elevated in Slf14 (Figure 3). Furthermore, *AP1*, a well-known global regulator of the antioxidant system, along with its AP1-like protein, *AP1L*, showed up-regulation in transcriptional activity, suggesting the presence of elevated ROS levels in Slf14. These findings imply significant differences in energy metabolism and redox states between Slf14 and Slf14(w), with Slf14 demonstrating a greater capacity for PQ synthesis.

### 2.4. qRT-PCR Analysis

To verify the results of transcriptome sequencing, a total of 15 genes were selected for qRT-PCR detection. These selected genes primarily pertain to the PQ biosynthesis (including *PKS*, *Omef*, *Hydro*, *FAD,* and *Zftf*), energy metabolism (encompassing *PDH*, *MDH*, *CS*, *HK*, and *PFK*), and redox processes (comprising *CAT*, *NOX*, *AP1L*, *GR*, and *APX*). The results indicated that the qRT-PCR findings were consistent with the transcriptome data (Figure 3B–D), thereby confirming the reliability of transcriptome data.

### 2.5. Measurement of Citric Acid and ATP Content

**Figure 4:**
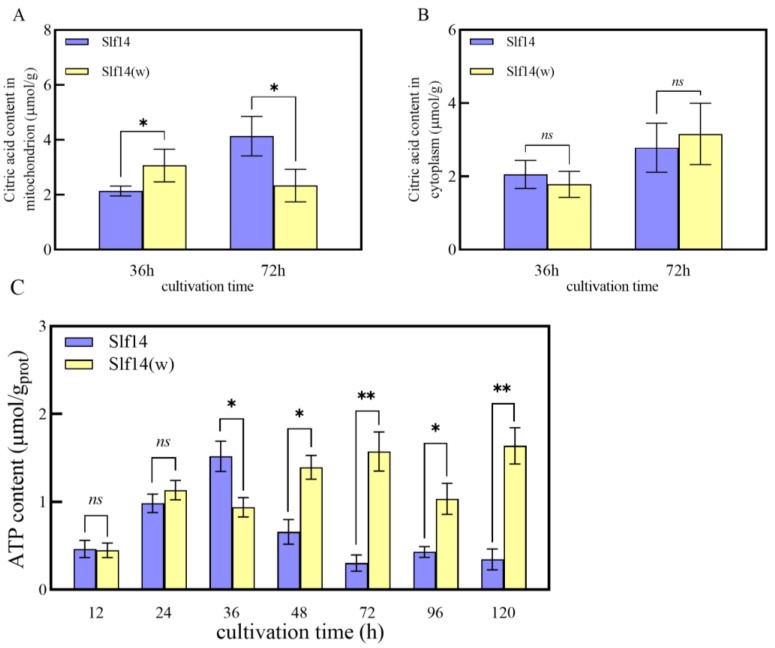
The changes in CA content and intracellular ATP in Slf14 and Slf14(w) cells at different time periods. Citric acid content in mitochondrial (**A**); Intracellular citric acid (**B**); Intracellular ATP in cytoplasm (**C**). Three biological replicates were carried out for each group of experiments, *, **, and ns indicating a significant difference *p* < 0.05, a very significant difference *p* < 0.01, and no significance, respectively.

The content of citric acid (CA) in the mitochondria and cellular matrix of the fungus was analyzed. The CA levels in the cytoplasm of strains Slf14 and Slf14(w) exhibited an increasing trend over time; however, no statistically significant difference was observed between the two strains (Figure 4A). In addition, the mitochondrial CA content of strain Slf14 significantly increased, while strain Slf14(w) experienced a notable decrease, resulting in the mitochondrial CA content of strain Slf14 being 1.77 times greater than that of strain Slf14(w) at 72 h (Figure 4B). 

ATP serves as the fundamental energy currency for all cellular processes and plays a pivotal role in regulating cellular metabolic functions [41,42]. During the initial 24 h of fermentation, there was virtually no difference in intracellular ATP content between strains Slf14 and Slf14(w). However, due to its highly efficient energy metabolism, Slf14 accumulated intracellular ATP during the initial phase of fermentation, reaching an intracellular ATP level of 1.52 ± 0.17 µmol/g protein at 36 h, which is 1.62 times that of Slf14(w). After 48 h, strain Slf14 initiated PQ biosynthesis, which consumes a significant amount of ATP, leading to a continuous decline in ATP levels. Conversely, strain Slf14(w) has a low capacity for PQ production, resulting in ATP synthesis exceeding consumption, and thus maintaining persistently high ATP levels in the fungal mycelia (Figure 4C). These findings further underscore that strain Slf14 has a higher aerobic metabolic flux than strain Slf14(w), providing a favorable physiological foundation for the substantial production of PQs in strain Slf14.

### 2.6. Effects of Addition of ROS-Related Reagents on PQ Production

**Figure 5:**
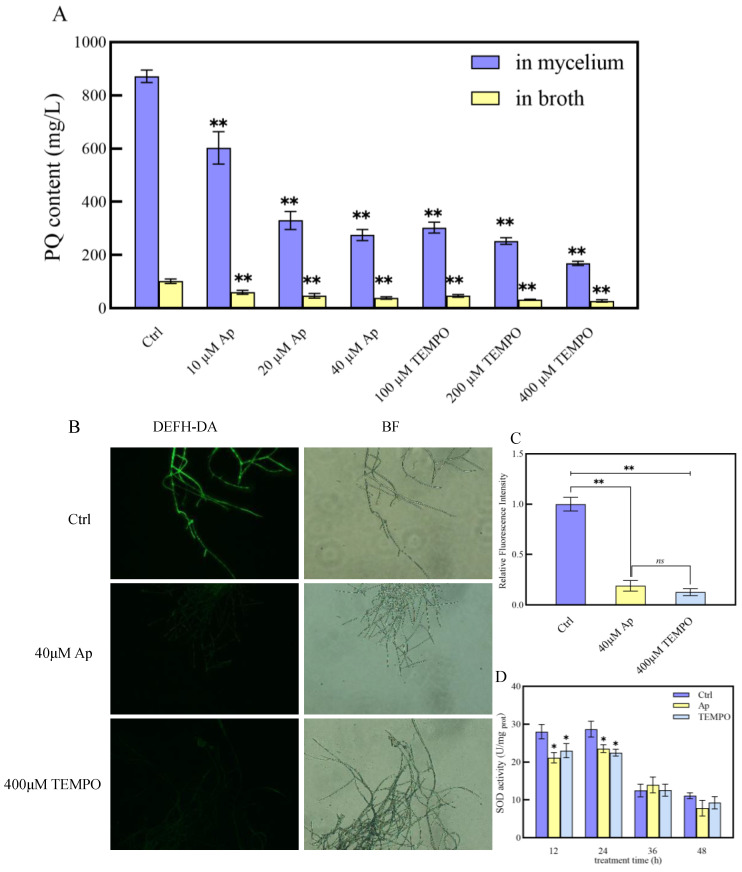
Effects of different concentrations of apocynin and TEMPO on *Shiraia* sp. Slf14 PQ yield (**A**). Effect of apocynin and TEMPO on intracellular ROS content of *Shiraia* sp. Slf14 (**B**). Original magnification: 400×. After the *Shiraia* sp. Slf14 mycelium was treated with 40 µM Ap (apocynin) and 400 µM TEMPO, the ROS fluorescence probe DCFH-DA was used to observe the change in ROS in the cell. BF was a bright field mode. The fluorescence intensity was quantified by image processing software ImageJ (Product version is 2006.02.01) (**C**). Changes in intracellular SOD enzyme activity after adding reagent (**D**). Ctrl is the control group. The experiment set up three independent repetitions. ns represents no significant difference, * represents a very significant difference from the control group (*p* < 0.05), and ** represents a very significant difference from the control group (*p* < 0.01).

**Figure 6:**
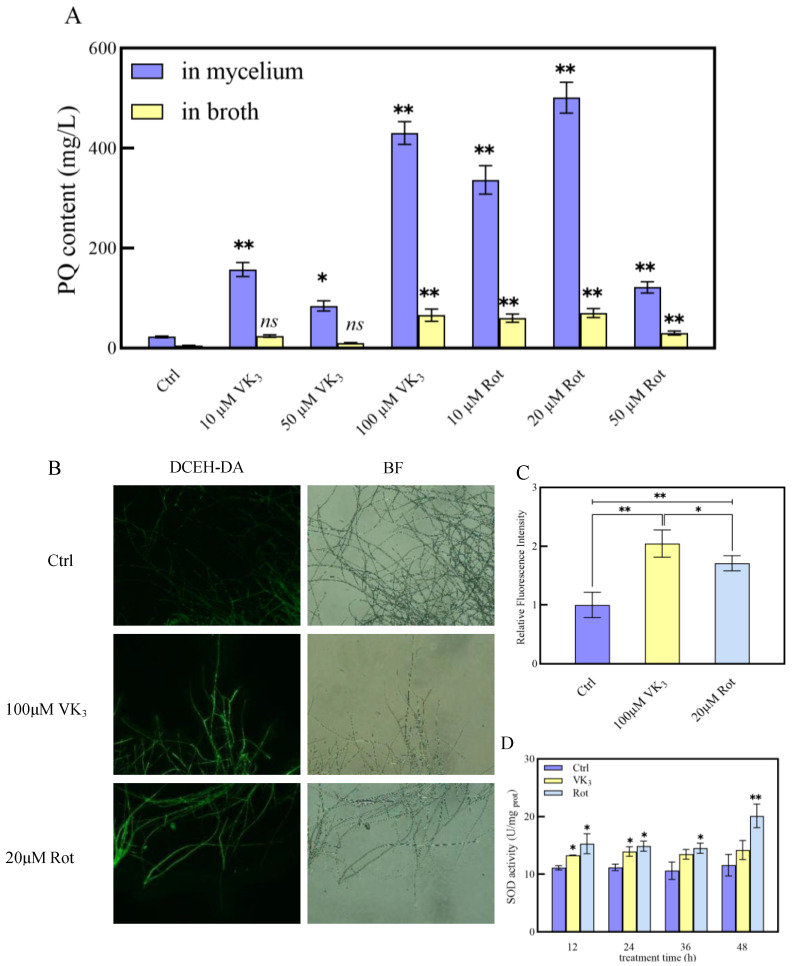
Effects of different concentrations of menadione and rotenone on *Shiraia* sp. Slf14(w) PQ yield (**A**). After the *Shiraia* sp. Slf14(w) mycelium was treated with 100 µM VK_3_(menadione) and 20 µM rotenone, the ROS fluorescence probe DCFH-DA was used to observe the change in intracellular ROS, and BF was a bright field mode (**B**). Original magnification: 400×. The fluorescence intensity was quantified by image processing software ImageJ (Product version is 2006.02.01) (**C**). Changes in intracellular SOD enzyme activity after adding reagent (**D**). Ctrl is the control group. The experiment set up three independent repetitions. ns represents no significant difference, * represents a very significant difference from the control group (*p* < 0.05), and ** represents a very significant difference from the control group (*p* < 0.01).

To elucidate the relationship between ROS and PQ biosynthesis, redox-related reagents were introduced into both strains. Upon the addition of 40 µM apocynin, an NADPH oxidase inhibitor, the Slf14 strain exhibited reduced intracellular and extracellular PQ contents of 275.13 ± 20.81 mg/L and 39.16 ± 4.32 mg/L, respectively, reflecting reductions of 68.45% and 61.69% compared to control conditions. Similarly, supplementation of Slf14 with 400 µM TEMPO, an ROS scavenger, resulted in intracellular and extracellular PQ contents of 169.33 ± 8.11 mg/L and 28.28 ± 4.32 mg/L, demonstrating reductions of 80.58% and 72.34% compared to control, respectively (Figure 5A). Additionally, levels of the ROS and SOD enzyme activity were significantly decreased in strain Slf14 treated with 40 µM apocynin and 400 µM TEMPO (Figure 5B–D), indicating these treatments effectively reduced oxidative stress levels in Slf14. Conversely, the exogenous addition of oxidative stress inducers rotenone (a mitochondrial complex I inhibitor) and menadione (a superoxide radical producer) led to significant increases in PQ production in strain Slf14(w). Specifically, the addition of 20µM rotenone resulted in the highest intracellular and extracellular PQ contents, measuring 501.17 ± 31.13 mg/L and 70.28 ± 9.22 mg/L, respectively, which corresponds to remarkable increases of 22.07- and 13.46-fold compared to the control, respectively. Meanwhile, the introduction of 100 µM menadione yielded intracellular and extracellular PQ contents of 430.55 ± 22.60 mg/L and 66.13 ± 12.33 mg/L, representing increases of 18.96- and 12.67-fold relative to the control, respectively (Figure 6A). In conclusion, levels of ROS and SOD enzyme activities were notably elevated in Slf14(w) upon exposure to 20 µM rotenone and 100 µM menaquinone, respectively (Figure 6B–D). Corresponding ROS-related reagents were added to each of the two strains, and the results visualized the effect of ROS on the production of PQs. All the aforementioned results indicate that ROS play a crucial role in enhancing PQ synthesis. Furthermore, the elevated oxidative state observed in Slf14 is a significant factor contributing to its elevated production of PQs.

### 2.7. Mitochondrial NADH/NAD^+^ Ratio

**Figure 7:**
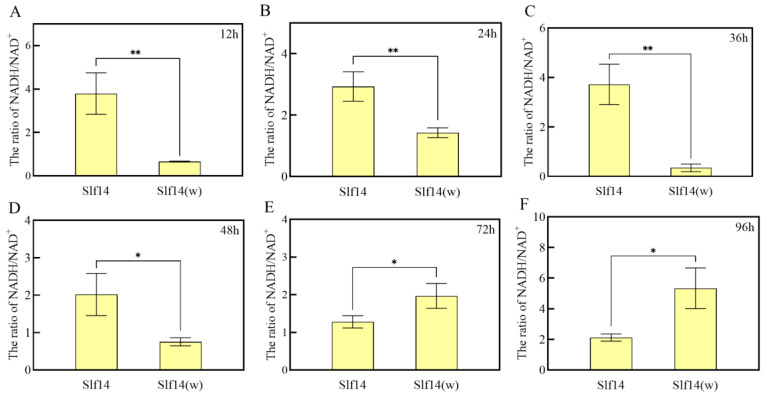
Changes in the intracellular NADH/NAD^+^ ratio between *Shiraia* sp. Slf14 and *Shiraia* sp. Slf14(w) at different time periods (**A**–**F**). Three biological replicates were carried out for each group of experiments, * indicates a significant difference *p* < 0.05 and ** indicates an extremely significant difference *p* < 0.01.

The dynamics of the NADH/NAD^+^ ratio were closely monitored, revealing a substantial difference between strains Slf14 and Slf14(w) (Figure 7). Notably, the NADH/NAD^+^ ratio of strain Slf14 was significantly higher than that of strain Slf14(w) prior to 48 h. For instance, at 36 h, the NADH/NAD^+^ ratio for Slf14 was measured at 3.72 ± 0.82, while Slf14(w) exhibited a ratio of 0.35 ± 0.15, highlighting a significant disparity (Figure 7C). Conspicuously, the intracellular NADH/NAD^+^ ratio in strain Slf14 decreased significantly after 36 h, whereas that of strain Slf14(w) notably increased. This resulted in a significantly lower NADH/NAD^+^ ratio for Slf14 compared to Slf14(w) from 72 to 96 h (Figure 7E,F). It is worth mentioning that, during this period, the H_2_O_2_ content in both strains transitioned from a highly significant difference to a non-significant difference (Figure 2A). These parallel changes in ROS levels and NADH/NAD^+^ ratios suggest that the NADH/NAD^+^ ratios are a pivotal factor influencing the oxidative state of the fungi.

### 2.8. Mitochondrial Complex Activity and Mitochondrial Membrane Potential Assays

**Figure 8:**
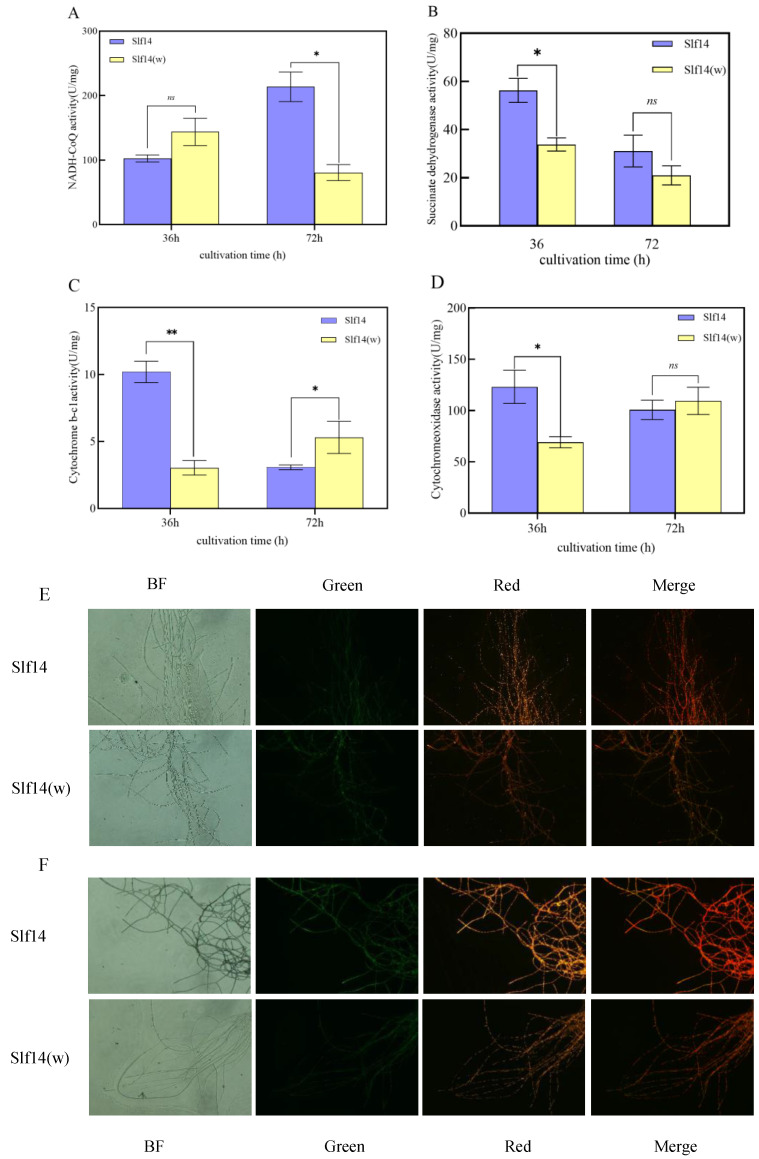
Mitochondrial complex activities and mitochondrial membrane potential of *Shiraia* sp. Slf14 and *Shiraia* sp. Slf14(w) at different time periods. The activity of NADH-CoQ (**A**), cytochrome b-c1activity (**B**), and Cytochromeoxidase activity (**C**). Mitochondrial membrane potential of Slf14 and Slf14(w) at 36 h (**D**). Mitochondrial membrane potential of Slf14 and Slf14(w) at 72 h (**E**,**F**). Original magnification: 400×. Three biological replicates were carried out for each group of experiments, * indicates a significant difference *p* < 0.05 and ** indicates an extremely significant difference *p* < 0.01, ns represents no significant difference.

The activity of complex I in strain Slf14 at 36 h was significantly lower than that observed at 72 h. In contrast, the activity of complex I in Slf14(w) was significantly higher at 36 h compared to 72 h (Figure 8A). This indicates that the enzyme activity of complex I is diminished at 36 h in strain Slf14. At 36 h, the activity of complex II in Slf14 was significantly greater than that of Slf14(w) during the same period (Figure 8B). This may result from the inhibition of complex I activity in Slf14 during this timeframe, with the succinate oxidative respiratory chain associated with complex II functioning efficiently to sustain a high level of energy metabolism in the strain. Similarly, the significant increase in the activities of complex III and IV at 36 h further supports this hypothesis. There were no significant differences in the activity of complex II between the two strains at 72 h (Figure 8B). At 72 h, the activity of complex III (Figure 8C) in Slf14 was significantly lower than that in Slf14(w), while no significant difference was observed in the activity of complex IV between the two strains (Figure 8D). 

Additionally, at 36 h, Slf14 exhibited a lower mitochondrial membrane potential (MMP) compared to Slf14(w) (Figure 8E). The reduced MMP suggests that an excess of intracellular ROS caused damage to the fungus. By 72 h, during the secondary metabolic production phase, the intracellular ROS levels in Slf14 were lower than at 36 h, and the cellular membrane potential had increased, returning to a normal level. In contrast, the membrane potential of Slf14(w) remained unchanged from its level at 36 h (Figure 8F). The alteration in the membrane potential profile indicates that Slf14 organisms can mitigate the damage caused by oxidative stress through the synthesis of PQs.

## 3. Discussion

Metabolism is fundamental to microbial life, underpinning all cellular functions by supplying the necessary energy and building blocks [43]. Primary metabolites, which are precursor molecules produced during growth, play a crucial role in maintaining cellular functions and serve as the raw materials for secondary metabolites and extracellular enzymes [44]. Notably, in the present study, residual sugars were significantly lower in Slf14 compared to Slf14(w) on the second day of fermentation, despite no significant difference in the biomass of the organisms. This discrepancy indicates that the primary energy metabolism in Slf14 organisms was more efficient, allowing for greater energy production for secondary products. The analysis of transcriptome data from both strains revealed that the expression of genes associated with energy metabolism, redox processes, and PQ synthesis was higher in Slf14 compared to Slf14(w). The elevated energy metabolism in Slf14 results in an increased accumulation of NADH, leading to a higher NADH/NAD^+^ ratio, which subsequently raises ROS levels. To mitigate ROS damage to fungi, Slf14 enters the PQ synthesis phase, during which the increased demand for precursors and ATP further enhances energy metabolism. This interaction establishes a positive feedback loop among energy metabolism, reactive oxygen species (ROS), and PQ synthesis. Exogenously added ATP has been shown to function as a signaling molecule that enhances hypocrellin A synthesis in the co-culture system of *Shiraia* sp. S9 and *Pseudomonas fulva* SB1 [45]. Additionally, the incorporation of glutathione has been demonstrated to improve the yield of the fermentation product 2-keto-L-glutamic acid in a co-culture system involving keto-based *K. vulgare* 25B-1 and *B. endophyticus* ST-1 [46]. These findings suggest a significant relationship between energy metabolism, ROS, and the production of secondary metabolites.

A well-established connection exists between the response to oxidative stress and the activation of secondary metabolism, as demonstrated by previous studies [47]. Increased oxidative stress results in enhanced lipid peroxidation and free radical production, which in turn promotes aflatoxin synthesis [48]. Furthermore, antioxidants can inhibit the expression of nearly all genes within the aflatoxin biosynthesis gene cluster, thereby suppressing aflatoxin synthesis [49]. Additionally, treatment with curcumin can lead to a dramatic increase in O_2_^−^ and H_2_O_2_ levels in *Aspergillus flavus*, causing a burst of ROS that reduces aflatoxin synthesis [50]. Salicylic acid can enhance GA synthesis by inhibiting ROS production from mitochondrial complex III activity, while heat stress can act on NOX to increase ROS levels, thereby improving GA production in *G. lucidum* [51,52]. In *Arabidopsis*, AtPER1 has been identified as an inhibitor of abscisic acid (ABA) catabolism and GA biosynthesis, functioning by eliminating ROS [53]. In the case of *Shiraia* spp., various stimuli, including light-dark displacement [54], low-intensity ultrasound [55], blue light [56], TX100 [57], and La^3+^ [40], have been shown to stimulate the production of HA through the generation of H_2_O_2_. Notably, the activities of redox-related enzymes and the H_2_O_2_ content in Slf14 were consistently higher than those in Slf14(w) during the pre-growth period (Figure 2), suggesting that Slf14 maintains a higher oxidation state. This elevated oxidation level triggers a stress response in the fungus, prompting the up-regulation of the gene cluster related to PQ biosynthesis and stimulating PQ biosynthesis. Moreover, the introduction of ROS-related chemicals into both Slf14 and Slf14(w) altered intracellular ROS levels, thereby regulating the accumulation of PQs (Figure 5A and Figure 6A). Therefore, our findings support the conclusion that the heightened intracellular oxidative state in Slf14 results in increased PQ production.

In fungi, the primary source of intracellular ROS production is the mitochondrion [51]. The mitochondrion generates high levels of ROS due to electron leakage from the Electron Transport Chain (ETC) complexes I and III [58,59]. Under certain conditions, such as when inhibited by antimycin, complex III can induce the production of superoxide (O_2_^·−^). However, under normal physiological conditions, its contribution to ROS production in the mitochondria is considerably lower than the maximum rate of O_2_^·−^ production by complex I and is typically negligible [60]. Additionally, ROS can be generated under specific conditions through the single electron reduction of oxygen via various oxidases, including NAD(P)H oxidase (NOX family) and xanthine oxidase (XO) [39,61,62]. In *G. lucidum*, NOX influences GA biosynthesis under water stress conditions by regulating ROS [51]. Likewise, the introduction of the NOX inhibitor apocynin led to a reduction in intracellular ROS levels in Slf14, which significantly decreased the PQ content of Slf14 (Figure 5A). This finding suggests that NOX also serves as a site for ROS production. Furthermore, when exposed to visible light, PQs can react with oxygen to form potent reactive oxygen species with high quantum efficiency [21]. Organisms adapt to redox stimuli by modulating the NADH/NAD^+^ ratio, which influences the rate of superoxide formation [63]. Inhibition of mitochondrial complex I activity disrupts the oxidation of NADH by the ETC in the mitochondria, resulting in a sustained high NADH/NAD^+^ ratio [64]. Notably, changes in the NADH/NAD^+^ ratio aligned with trends in fungal ROS levels (Figure 2 and Figure 7), and the activity of complex I in both strains corresponded with the trends in ROS levels. These findings suggest that complex I and NOX serve as the primary sites of ROS production in both strains and that the NADH/NAD^+^ ratio is a critical factor influencing the oxidative state of the strains. The NADH/NAD^+^ ratio is intricately linked to mitochondrial complex I activity and energy metabolism.

The synthesis of secondary products often necessitates energy and intermediates derived from primary metabolism [65]. In fungi, the TCA cycle occurs in the mitochondria and functions as a critical metabolic pathway with dual roles: energy production and the generation of anabolic precursors [66]. At the stage of PQ synthesis, the intracellular ATP content in Slf14 was significantly lower than that in Slf14(w) (Figure 4C), indicating that PQ synthesis demands substantial energy consumption. Research has demonstrated that mitochondrial pyruvate carriers influence GA synthesis by modulating metabolic flux, and the addition of acetyl coenzyme A produced through fatty acid oxidation has been shown to enhance GA biosynthesis in *G. lucidum* [67]. Notably, at 36 h, the TCA cycle rate in Slf14 exceeded that of Slf14(w), yet the mitochondrial content of CA was significantly lower in Slf14 compared to Slf14(w). Acetyl coenzyme A acts as a common precursor for both CA and PQs. During this period, an increased influx of acetyl coenzyme A into PQ synthesis resulted in a decreased CA content in the mitochondria of Slf14 relative to Slf14(w). By 72 h, however, the CA content in the mitochondria of Slf14 was significantly higher than that in Slf14(w), accompanied by elevated extracellular CA levels. A portion of the accumulated CA, generated via aerobic metabolism, was utilized in the TCA cycle, while the remainder crossed the mitochondrial membrane and entered the cytoplasm through the TCA cycle transport system. Concurrently, *ACL* expression in Slf14 was significantly up-regulated. With the presence of highly active ACL, cytoplasmic CA generated acetyl coenzyme A, a precursor essential for PQ synthesis. The high activity of enzymes associated with energy metabolism, along with the accumulation of CA, suggests that Slf14 exhibits enhanced metabolic activity, thereby providing an ample supply of energy and precursor molecules for PQ synthesis.

Furukawa et al. [68] made a significant discovery, highlighting the critical role of mitochondrial energy metabolism in aflatoxin synthesis. They found that the degradation of components associated with mitochondrial energy could effectively suppress aflatoxin production. This synthesis appears to be closely linked to the energy status of subcellular structures. Inhibition of energy metabolism in *R. oryzae* may lead to a reduction in its growth [69]. At 36 h, Slf14 exhibited inhibited complex I activity, an abnormal mitochondrial membrane potential (Figure 8E), and impaired mitochondrial function. Interestingly, the intracellular ATP content of Slf14 at this stage was significantly higher than that of Slf14(w). This seemingly paradoxical observation can be explained by the inhibition of complex I activity, which suppresses the NADH oxidative respiratory chain. To meet the organism’s energy needs, there is an accelerated transfer of electrons to complex III and complex IV, stemming from the FADH_2_ oxidative respiratory chain, resulting in a marked increase in the activities of complex III and complex IV in Slf14 at 72 h compared to Slf14(w) (Figure 8C,D). By 72 h, complex I activity had returned, mitochondrial function was restored (Figure 8F), and the activities of complex III and IV had normalized. ATP serves as the driving force for all cellular functions and acts as a crucial regulator of cellular metabolism [41,42]. ATP and energy charge are essential for various physiological processes [70]. In this study, Slf14 accumulated a substantial amount of intracellular ATP during the pre-synthesis period, which could provide energy for subsequent PQ synthesis and serve as a signaling molecule to stimulate PQ synthesis. Upon entering the PQ synthesis phase, the intracellular ATP levels in Slf14 were maintained at a low level, which in turn initiated a positive feedback loop within the energy metabolism cycle of Slf14. Consequently, it is reasonable to speculate that the increased energy metabolism of Slf14 not only raises ROS levels to promote PQ synthesis in fungal cells but also supplies a considerable amount of energy and precursor molecules, thereby establishing a physiological foundation for its transition into secondary metabolism for the mass production of PQs.

## 4. Materials and Methods

### 4.1. Fungal Strains and Culture Conditions

The strain *Shiraia* sp. Slf14 (CCTCC M209294) utilized in this study is an endophytic fungus associated with *Huperzia serrata* [33], whereas *Shiraia* sp. Slf14(w) (CCTCC M20211702) represents its low-PQ-producing mutant strain [36]. To maintain these fungal strains, they were inoculated into 20% glycerol tubes and stored at −80 °C. The complete genome of *Shiraia* sp. Slf14 has been sequenced, with the accession number AXZN00000000 [34]. For submerged fermentation, the strains Slf14 and Slf14(w) were first inoculated from a slant at 4 °C onto potato fructose agar (PFA; 20 g/L fructose, 200 g/L potato extract, and 18 g/L agar) plates and incubated at 28 °C for 7 days. Subsequently, mycelia were harvested from the plates and incubated in a 500 mL conical flask containing 140 mL of seed medium (20 g/L fructose, 200 g/L potato extract) at 28 °C and 150 rpm for 3 days to prepare the seed broth. Following this, 8 mL of the seed broth was transferred to a fermentation medium (PFB; 60 g/L fructose, 200 g/L potato extract, 5 g/L yeast extract) and cultivated at 28 °C and 160 rpm for 8 days [71].

### 4.2. Analysis of Cell Growth and Residual Sugar

Biomass accumulation was assessed through dry cell weight (DCW) analysis. Three parallel samples were collected from various treatments at different time points, filtered through a 200-mesh sieve, and washed three times with ultrapure water. The fungal mycelium was then dried in an oven at 50 °C until a constant weight was achieved and subsequently weighed [7]. To determine the residual sugar in the fermentation broth, the 3,5-dinitrosalicylic acid (DNS) method was employed [72]. After performing a 10-fold dilution of the fermentation solution, 1 mL of the fermentation broth was pipetted into a 25 mL colorimetric tube, with ultrapure water serving as a control. Next, 1 mL of ultrapure water and 2 mL of DNS solution were added to each tube, which was then shaken thoroughly and placed in boiling water for 10 min. Following this, the reaction solution was removed, cooled to room temperature, and diluted to a final volume of 25 mL with ultrapure water. The absorbance of fructose was measured at 540 nm using a UV-1800 (PC) UV-Vis spectrophotometer (Mapada, Shanghai, China), and the concentration of the remaining fructose was calculated based on the fructose standard curve.

### 4.3. Extraction and Analysis of Total PQs

To determine intracellular PQs, dried mycelium was pulverized into a fine powder using a grinder. Subsequently, 1.0 g of the mycelium powder was weighed and combined with 100 mL of acetone for Soxhlet extraction at 55 °C, continuing until the reflux solution became colorless. Concurrently, extracellular PQs were extracted three times by mixing 20 mL of fermentation broth with an equal volume of dichloromethane. The collected intracellular and extracellular pigments were evaporated at 45 °C using a reduced-pressure rotary evaporator, after which the residue was dissolved in 10 mL of acetonitrile and filtered through 0.22 µm nylon filtration membranes (Millipore, Darmstadt, Germany). The total PQs were quantified using high performance liquid chromatography (HPLC, Waters 2996 system, Milford, MA, USA) [8,71]. The chromatographic conditions employed included a YMC Triart C18 column (250 mm × 4.6 mm, 5 µm, YMC Co,. Ltd., Tokyo, Japan), a column temperature of 35 °C, a solvent ratio of acetonitrile to water of 7:3, a flow rate of 1 mL/min, and a detection wavelength of 460 nm [8].

### 4.4. Determination of H_2_O_2_ Content and Various Oxidoreductases

Fungal organisms from various periods were collected and washed twice with ultrapure water for subsequent index determinations. Superoxide dismutase (SOD) activity was assessed using the nitro tetrazolium chloride blue (NBT) method [31], wherein the superoxide anion reacts with yellowish NBT to produce blue diformazan, exhibiting a maximum absorption peak at 560 nm. The presence of SOD mitigates the O_2_^−^ generated by the photoreduction of riboflavin, allowing the magnitude of the SOD enzyme activity to be inferred from the intensity of the blue color in the reaction system. NBT staining was utilized to visualize ROS within the fungal organisms. Mycelia cultured in shaking flasks over varying periods were collected, and an appropriate quantity of organisms was mixed with 2 mL of 0.5 mg/mL NBT staining solution. The mixture was stained for 1 h under light conditions before being transferred to slides for microscopic observation. Catalase (CAT) activity measurements were performed using a commercially available catalase assay kit (A007-1-1, Nanjing Jiancheng Bioengineering Institute, Nanjing, China), where one unit of activity (U) is defined as the decomposition of 1 µmol of H_2_O_2_ per milligram of histone per second. H_2_O_2_ content was quantified using an H_2_O_2_ kit (A064-1-1, Nanjing Jiancheng Bioengineering Institute, Nanjing, China), and the content was calculated according to the provided formula. Glutathione peroxidase (GPX) activity was measured using the GPX kit (A005-1-2, Nanjing Jiancheng Bioengineering Institute, Nanjing, China), with GPX activity expressed as the reaction rate of catalyzed GSH. The protein concentration in the suspension was determined by the Bradford method [42], using bovine serum albumin (BSA) as a standard.

### 4.5. Transcriptome Sequencing, Annotation, and Analysis

Strains fermented for 36 h and 72 h were selected for sequencing. Slf14 served as the experimental group, while Slf14(w) from the same fermentation period acted as the control group. Mycelia collected at different time points were washed twice with ultrapure water. Total RNA was extracted from the mycelium using TRIzol reagent (Invitrogen, Carlsbad, CA, USA) following the manufacturer’s instructions. The extracted total RNA was subsequently treated with RNA-free DNase I (TaKaRa, Dalian, China). The enriched mRNA was fragmented and reverse transcribed into complementary DNA (cDNA), which was then purified and end-repaired. The cDNA library was sequenced and analyzed using the Illumina HiSeq 4000 platform (Majorbio, Shanghai, China). The raw data have been deposited in the China National GenBank Database (CNGBdb) with accession number CNP0006025. Raw sequencing data files were trimmed and filtered using Sickle (https://github.com/najoshi/sickle (accessed on 21 June 2018)) and SeqPrep (https://github.com/jstjohn/SeqPrep) tools to obtain clean reads for subsequent analysis. The clean reads were aligned with the reference genome using HiSat2 (http://ccb.jhu.edu/software/hisat2/index.shtml (accessed on 21 June 2018)), and the overall quality of the alignment results was assessed. Using BlastX (E-value < 10^−5^), single gene sequences obtained from de novo assembly were compared with the NCBI NR (Non-redundant) protein, Swiss-Prot, Pfam (Protein Family Database), STRING (Search Tool for Recurring Instances of Neighbouring Genes, http://ccb.jhu.edu/software/stringtie/ (accessed on 21 June 2018)), GO (Gene Ontology Consortium), and KEGG (Kyoto Encyclopedia of Genes and Genomes) databases.

Gene functions were annotated using the NR protein database (https://www.ncbi.nlm.nih.gov (accessed on 21 June 2018)), Swiss-Prot (https://www.expasy.ch/sprot (accessed on 21 June 2018)), KEGG (https://www.genome.jp/kegg (accessed on 21 June 2018)), and the Gene Ontology (GO) database (https://geneontology.org (accessed on 21 June 2018)). Differential expression analysis was conducted using DEGseq software (version 1.26.0)), which evaluates read count data for the genes under comparison based on a negative binomial distribution model. The criteria for identifying significant differential gene expression included a false discovery rate (FDR) < 0.05 or |log_2_^FC^| ≥ 1.

### 4.6. Quantitative Real-Time PCR (qRT-PCR) Analysis of Gene Expression

Mycelia collected at 72 h were washed twice with ultrapure water, and total RNA was extracted using TRIzol reagent (Invitrogen, Carlsbad, CA, USA), followed by treatment with RNA-free DNase I (TaKaRa, Dalian, China). The extracted total RNA was diluted to a uniform concentration and subsequently processed according to the ReverTra HiScript III RT SuperMix for qPCR (+gDNA wiper) Reverse Transcription Kit (Toyobo, Osaka, Japan). The primers utilized in this study are detailed in Supplementary Materials Table S2. The qRT-PCR was conducted on an ABI 7500 real-time PCR system (Applied Biosystems, Foster City, CA, USA) using the Taq Pro Universal SYBR qPCR Master Mix Fluorescence Quantification Kit (TaKaRa, Dalian, China) in a reaction volume of 20 µL. The PCR preconditioning protocol included pre-denaturation at 95 °C for 10 min, denaturation at 95 °C for 10 s, annealing at 55 °C for 30 s, and extension at 60 °C for 34 s, followed by 40 cycles of amplification (95 °C for 15 s and 60 °C for 34 s). The expression levels of the relevant genes were determined through relative quantification, with Slf14 serving as the experimental group and Slf14(w) as the control group. GAPDH was selected as the internal reference gene, and the results were analyzed using the 2^−ΔΔCT^ method [9].

### 4.7. Determination of the Contents of Citric Acid and ATP

The intracellular citric acid (CA) content was quantified using a CA content assay kit (BC2150, Solarbio, Beijing, China). ATP is essential for providing energy during the luciferase-catalyzed production of fluorescent light from fluorescein, with ATP content being directly proportional to fluorescence intensity within a specific range. Consequently, this method enables the sensitive detection of intracellular ATP levels. Approximately 20 mg of mycelium from various growth periods was placed in a homogenizer, to which 200 µL of lysate was added and thoroughly homogenized in an ice bath. The resulting supernatant was centrifuged at 12,000 rpm for 5 min at 4 °C and was subsequently utilized for further analysis. The intracellular ATP content was measured using the Enhanced ATP Assay Kit (S0027, Beyotime, Shanghai, China).

### 4.8. Effect of Oxidative Stress Reagents Addition on PQ Biosynthesis

To investigate the impact of oxidative stress on PQ biosynthesis, various concentrations and types of oxidative stress reagents were introduced within 12 h after culturing the strains. The ROS synthesis facilitator menadione and the mitochondrial complex I inhibitor rotenone [73] were added to Slf14(w), while the NADPH oxidase inhibitor apocynin and the ROS scavenger TEMPO (2,2,6,6-tetramethylpiperidin-1-oxyl) [74] were added to Slf14, respectively. The intracellular ROS content was measured using the fluorescent probe 2,7-dichlorofluorescein diacetate (DCFH-DA) (S0033S, Beyotime, Shanghai, China). Under varying experimental conditions, a small amount of mycelium incubated for 1–2 days was collected and placed on a coverslip. The mycelium was washed three times with phosphate-buffered saline (PBS) buffer, and a diluted staining solution of DCFH-DA at a final concentration of 10 µM was added. The samples were incubated for 30 min at 28 °C in the absence of light, after which the excess fluorescent staining solution was removed by washing with PBS buffer. Changes in intracellular ROS levels were observed using an inverted fluorescence microscope (Eclipse Ti-s, Nikon, Tokyo, Japan) following the different treatments. Fluorescence intensity changes were quantitatively analyzed using ImageJ software (NIH, Bethesda, MD, USA, Product version is 2006.02.01), with the average fluorescence intensity calculated as the total fluorescence intensity of all mycelia divided by the number of mycelia in the image [75]. Fermentation was conducted at 28 °C and 160 rpm in shake flasks for 7 days, after which the content of PQ was determined.

### 4.9. Determination of the NADH/NAD^+^ Ratio in Fungi

Appropriate amounts of fresh organisms were collected and washed three times with pre-cooled PBS, before being transferred to a glass homogenizer. Subsequently, 400 µL of NAD^+^/NADH extract was added, and the mixture was homogenized in an ice bath. The sample was then centrifuged at 12,000 rpm for 10 min at 4 °C, and the supernatant was collected for measurement. A portion of the sample was heated at 60 °C in a water bath for 30 min to decompose the NAD^+^ present in the mycelium. In a separate procedure, 20 µL of the organisms to be tested, along with samples subjected to water bath treatment, were added to a 96-well plate. To this, 90 µL of ethanol dehydrogenase working solution was incorporated, mixed thoroughly, and incubated at 37 °C in the dark for 10 min. Following this, 10 µL of color development solution was added, mixed well, and incubated at 37 °C for 30 min under dark conditions, after which the absorbance was measured at 450 nm. The NADH content was determined for the water bath-treated samples, while the total NAD^+^ content was assessed in the untreated samples. The intracellular levels of NAD^+^ and NADH in the organisms were quantified using the WST-8 method NAD^+^/NADH assay kit (S0175, Beyotime, Shanghai, China).

### 4.10. Assay of Mitochondrial Complex Activity and Detection of Mitochondrial Membrane Potential

The collected mycelium was washed twice with ultrapure water and ground using a sterilized mortar and pestle. A 0.1 g sample of mycelium powder was homogenized with 1 mL of extract in an ice bath and then centrifuged at 600× *g* for 10 min at 4 °C. The supernatant was transferred to another centrifuge tube and subjected to a second centrifugation at 11,000× *g* at 4 °C; the resulting precipitate was kept on ice for subsequent analysis of the indexes. The activity of the mitochondrial respiratory chain complex I was evaluated using a kit (BC0510, Solarbio, Beijing, China), with one unit of enzyme activity defined as the consumption of 1 nmol of NADH per minute per mg of histone. Mitochondrial complex II activity was measured using the SDH activity assay kit (BC0950, Solarbio, Beijing, China), where one unit of enzyme activity (U) was defined as the consumption of 1 nmol of substrate per minute per milligram of protein. Complex III activity was assessed with a kit (BC3240, Solarbio, Beijing, China), defining one unit of enzyme activity as the catalytic production of 1 nmol of reduced cytochrome C per minute per mg of histone. Complex IV activity was determined using a kit (BC0945, Solarbio, Beijing, China), with one unit of enzyme activity defined as the degradation of 1 nmol of reduced cytochrome C per minute per mg of histone.

Mitochondrial membrane potential (MMP) is a critical component of proton movement dynamics and serves as a significant indicator for assessing mitochondrial function. A small quantity of mycelia cultured in liquid for varying durations was placed on a coverslip, and 1 mL of JC-1 staining working solution was added to cover the mycelia. The sample was incubated for 20 min at 28 °C in a temperature-controlled oven protected from light. Following incubation, the mycelium was washed 2 to 3 times with pre-cooled JC-1 buffer, and changes in the mitochondrial membrane potential of the mycelium were observed using fluorescence microscopy (Eclipse Ti-s, Nikon, Tokyo, Japan).

### 4.11. Bioinformatics Analysis and Statistical Analysis

The bioinformatics analysis focused on the genes within the genome of *Shiraia* sp. Slf14 (AXZN00000000). All determinations were performed in triplicate, and the results are presented as mean ± standard deviation (SD). Data analysis was conducted using IBM SPSS statistical software (Armonk, New York, NY, USA, Product version is 20.0.0.0). Statistical significance was assessed using one-way ANOVA followed by a *t*-test, with *p* < 0.05 considered indicative of a statistically significant difference.

## 5. Conclusions

**Figure 9:**
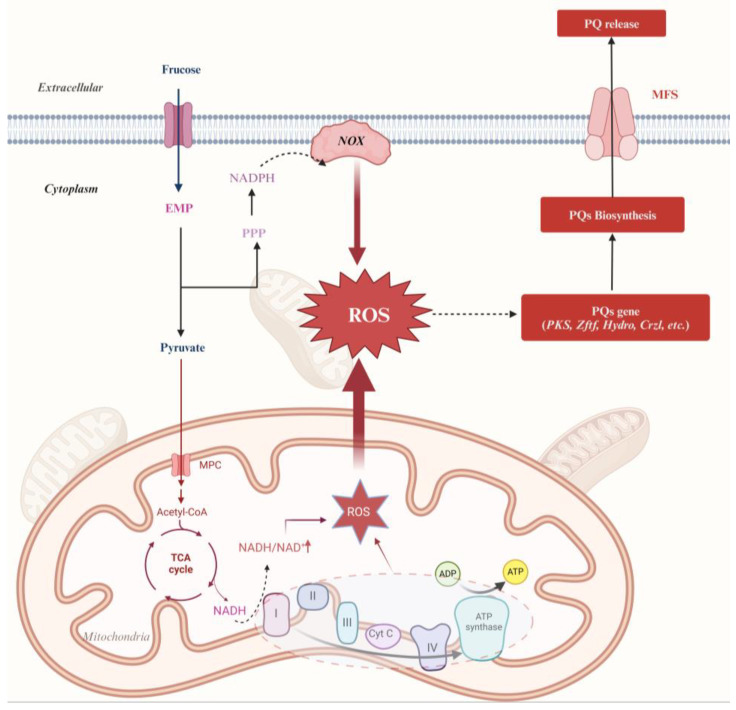
Schematic representation showing that the energy metabolism is controlled by ROS and the biosynthesis of PQ. NADH/NAD^+^ and *NOX* induce *Shiraia* sp. Slf14 to synthesize ROS. The solid line indicates the pathways that have been identified in this study, and the dotted line indicates what was confirmed in other studies and pathways not identified in this study. *NOX*, NADPH oxidase; *Hydro*, hydroxylase; *PKS*, polyketide synthase; *Crzl*, calcineurin-responsive zinc finger transcription factor; *Zftf*, zinc finger transcription factor; *MFS*, MFS transporters. Created with BioRender.com.

In sum, our study has demonstrated that Slf14 exhibited a higher metabolic rate compared to Slf14(w). This heightened energy metabolism facilitated the accumulation of significant amounts of NADH in Slf14. The increased levels of NADH accelerated both electron transfer and ATP synthesis within ETC. However, this acceleration also resulted in enhanced electron leakage from the ETC, leading to elevated levels of ROS, which can be detrimental to fungi at such high concentrations. Subsequently, the fungus entered the PQ biosynthesis phase, which effectively mitigated the damage caused by elevated oxidative levels on the mycelium, while concurrently reducing the NADH/NAD^+^ ratio of Slf14. During this phase, the carbon flux within energy metabolism shifted towards PQ synthesis (Figure 9). A considerable amount of ATP and certain primary metabolic intermediates were directed toward PQ biosynthesis, thereby maintaining subsequent ATP levels in Slf14 at a low level. The reduced ATP concentration further stimulated the cycling rate of the EMP and TCA cycles, marking the point at which the fungus established a positive feedback loop in energy metabolism. This loop reinforced its ability to thrive and produce PQs effectively, even in the presence of heightened oxidative stress. Collectively, our findings offer a valuable strategy for large-scale PQ production and contribute to a deeper understanding of ROS signaling in the biosynthesis of fungal metabolites.

## Data Availability

The raw data have been deposited in the China National GenBank Database (https://db.cngb.org/) with accession number CNP0006025. The other referenced data are included in the article or the Supplementary Materials.

## Supplementary Materials

The following supporting information can be downloaded at: https://www.mdpi.com/article/10.3390/ijms251810113/s1.

## Abbreviations

ABC	ATP-binding cassette
ACO	Aconitase
APX	Aseorbateperoxidase
AP1L	AP1-like transcription factor
BSA	bovine serum albumin
CA	citric acid
CAT	Catalase
Crzl	Calcineurin-responsive zinc finger transcription factor
CS	Citrate synthase
DCFH-DA	2,7-dichlorofluorescein diacetate
DCW	Dry cell weight
DNS	3,5-dinitrosalicylic acid
ETC	Electron Transport Chain
FAD	FAD/FMN-dependent oxidoreductase
GR	Glutathione reductase
GPX	Glutathione peroxidase
HA	Hypocrellin A
HK	Hexokinase
H_2_O_2_	Hydrogen peroxide
Hydro	Hydroxylase
MMP	mitochondrial membrane potential
MDH	Malate Dehydrogenase
MFS	MFS transporters
NOX	NADPH oxidase
NBT	Nitrotetrazolium blue chloride
Omef	O-methytransferase
PFA	Potato fructose agar
PFB	Potato fructose broth
PDH	Pyruvate dehydrogenase
PFK	Phosphofructokinase
PKS	Polyketide synthase
PQ	Perylenequinones
PBS	Phosphate-buffered saline
ROS	Reactive oxygen species
SDH	Succinate dehydrgenase
SOD	Superoxide dismutase
TEMPO	2,2,6,6-tetramethylpiperidin-1-oxyl
Zftf	Zinc finger transcription
α-KGDH	*α*-ketoglutarate dehydrogenase
