# Supplementary material for: Energy Metabolism Enhance Perylenequinone Biosynthesis in Shiraia sp. Slf14 through Promoting Mitochondrial ROS Accumulation
Source: Int J Mol Sci. 2024 Sep 20;25(18):10113. doi: 10.3390/ijms251810113 (PMC11432641; doi:10.3390/ijms251810113)
Supplement: Supplementary file 1 [file ijms-25-10113-s001.zip › ijms-3189294-supplementary.pdf]

Table S1. Sequencing data statistics

Samples	*Shiraia* sp. Slf14	*Shiraia* sp. Slf14(w)
Raw reads	4.96×10^7	4.30×10^7
Raw bases	7.49×10^9	6.49×10^9
Clean Reads	4.92×10^7	4.24×10^7
Clean bases	7.37×10^9	6.34×10^9
Error%	0.0248	0.0252
Q20	98.14	97.94
Q30	94.33	93.91
GC content%	51.21	51.83

Table S2. Quantitative Real-time PCR primer

Target gene	Primer name	Primer sequence (5'-3')
GAPDH	GAPDH-F	ACGAGGAGATCAAGGAG
	GAPDH-R	GATGGAAGAGCGAGTGT
PKS	PKS-F	GATGCGTGATGAACTGGAATTAG
	PKS-R	TTGCTTCGGACTCGGTAGG
Omef	Omef-F	GACTGGTCGGACAAGTATGC
	Omef-R	GGCTCTGGCACAATGACTTC
FAD	FAD-F	TGCCGTCTGCTGGTCTTG
	FAD-R	ACACCAACGCCACCTACTC
zftf	zftf-F	CCTGGGCGAACTGAAAGA
	zftf-R	AGCCTGGACATTGTTGGAAG
Hydro	Hydro-F	TCTACACCGATGAAGACTACAATG
	Hydro-R	TCAGCCGAAGTCCAGAGAAC
CAT	CAT-F	CATTGCCGATGGAACTGT
	CAT-R	TGGATACAAGGTGGACAGC
NOX	NOX2-F	GGGTTCGCTTATTTGGCTGA
	NOX2-R	GCGTGAAGGGATGGTACTGC
AP1L	AP1L-F	GAATCTCCCTCCTCCTCA
	AP1L-R	ACACACATAACCCTCCGT
GR	GR-F	TGTTGTTTTCTCCCACCCT
	GR-R	CGACGACCTTTTCTTCCTC
APX	APX-F	TGCCCACTGACATGGAAAT
	APX-R	GCTGAGCGAAAGGAACACC
PDH	PDH-F	TTCGGCGAGAAGCGAGTGATT
	PDH-R	GTGTGTCTTTGCGGCGGAGTT
MDH	MDH-F	GCACCTTCTCGTCGTCT
	MDH-R	GGTAACCTCGCTCTTGG
CS	CS-F	ACAAGGATGGCAAGGTT
	CS-R	GTAGGGCGAGGAGAGAG
HK	HK-F	TGCATTTTTGGTACTGGTTG
	HK-R	GAGGACTTTGTGCTCGTTGT
PFK	PFK-F	AGATTACACCCCAACAGG
	PFK-R	GCATACAAATGACAGGAC

Figure S1. Volcano map of differentially expressed genes. The horizontal coordinate is the differential gene expression multiplicity between the experimental group and the control group, and the vertical coordinate is the FDR (False Discovery Rate), which is obtained by correcting for the significance of the difference p-value (p-value), with a smaller FDR indicating a larger significant difference. Each triangle and circle in the graph corresponds to a gene, with red and yellow indicating up-regulated genes, where yellow points are significant up-regulated genes with 0.01 < FDR < 0.05, and red is highly significant up-regulated genes with FDR < 0.01. Dark blue and light blue indicate down-regulated genes, where light blue dots are significant down-regulated genes with 0.01 < FDR < 0.05 and red are highly significant down-regulated genes with FDR < 0.01. Black dots are non-significant up- and down-regulated genes.

Figure S2. GO enrichment bubble diagram of differentially expressed genes between *Shiraia* sp. Slf14 and *Shiraia* sp. Slf14(w). X axis represents the name of GO Term; the Y axis represents the enrichment factor. * indicates significant difference $p < 0.05$, ** indicates extremely significant difference $p < 0.01$. *** indicates extremely significant difference $p < 0.001$.

Figure S3. KEGG enrichment bubble diagram of the differentially expressed gene between *Shiraia* sp. Slf14 and *Shiraia* sp. Slf14(w). X axis repressents the name of KEGG Pathway; the Y axis represents the enrichment factor. * indicates significant difference $p < 0.05$, ** indicates extremely significant difference $p < 0.01$. *** indicates extremely significant difference $p < 0.001$.

Figure S1.

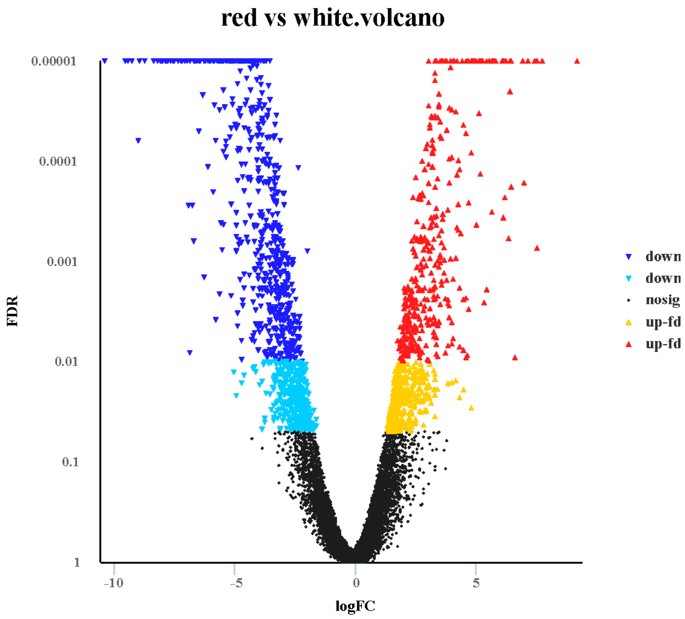

Figure S2.

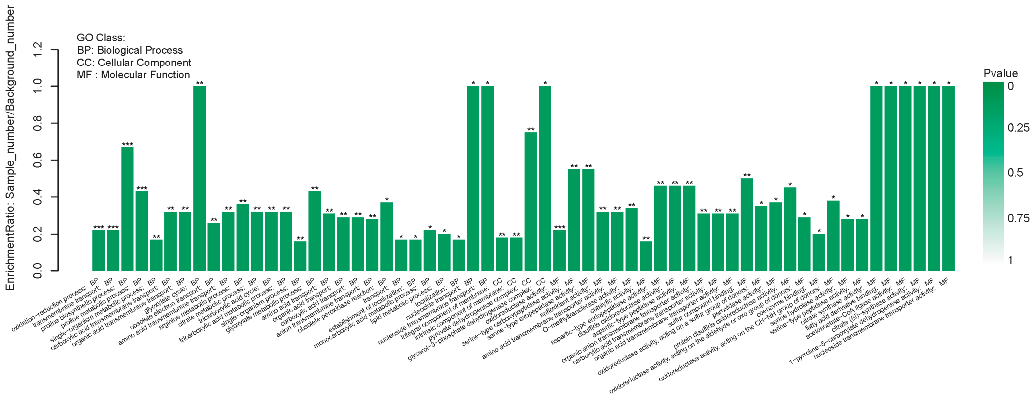

Figure S3.

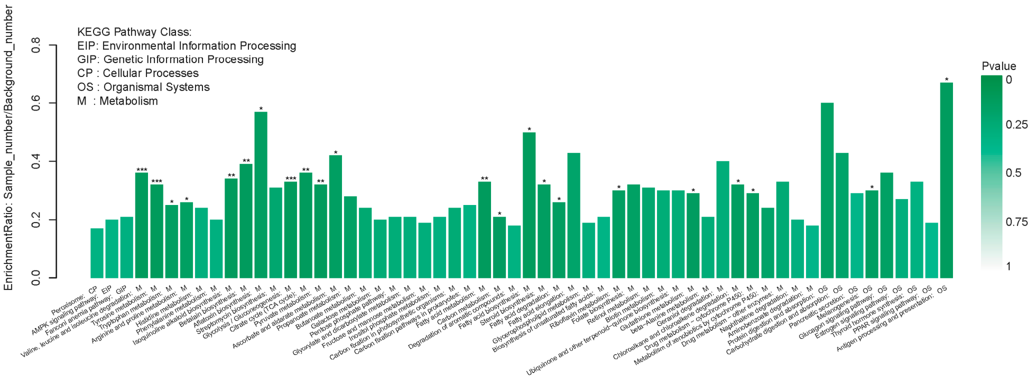

KEGG Pathway Class:
EIP: Environmental Information Processing
GIP: Genetic Information Processing
CP : Cellular Processes
OS : Organismal Systems
M : Metabolism
EnrichmentRatio: Sample_number/Background_number
0.0
0.2
0.4
0.6
0.8
Pvalue
0
0.25
0.5
0.75
1
Peroxisome: CP
AMPK signaling pathway: EIP
Fanconi anemia pathway: GIP
Valine, leucine and isoleucine degradation: M
Tyrosine metabolism: M
Tryptophan metabolism: M
Arginine and proline metabolism: M
Histidine metabolism: M
Phenylalanine metabolism: M
Isoquinoline alkaloid biosynthesis: M
Betalain biosynthesis: M
Aflatoxin biosynthesis: M
Streptomycin biosynthesis: M
Glycolysis / Gluconeogenesis: M
Citrate cycle (TCA cycle): M
Pyruvate metabolism: M
Ascorbate and aldarate metabolism: M
Propanoate metabolism: M
Butanoate metabolism: M
Galactose metabolism: M
Pentose phosphate pathway: M
Glyoxylate and dicarboxylate metabolism: M
Fructose and mannose metabolism: M
Inositol phosphate metabolism: M
Carbon fixation in photosynthetic organisms: M
Carbon fixation pathways in prokaryotes: M
Fatty acid metabolism: M
Carbon metabolism: M
Degradation of aromatic compounds: M
Fatty acid biosynthesis: M
Steroid biosynthesis: M
Fatty acid degradation: M
Fatty acid elongation: M
Glycerophospholipid metabolism: M
Biosynthesis of unsaturated fatty acids: M
Riboflavin metabolism: M
Folate biosynthesis: M
Retinol metabolism: M
Biotin metabolism: M
Ubiquinone and other terpenoid−quinone biosynthesis: M
Glutathione metabolism: M
beta−Alanine metabolism: M
Geraniol degradation: M
Chloroalkane and chloroalkene degradation: M
Drug metabolism − cytochrome P450: M
Metabolism of xenobiotics by cytochrome P450: M
Drug metabolism − other enzymes: M
Naphthalene degradation: M
Aminobenzoate degradation: M
Protein digestion and absorption: OS
Carbohydrate digestion and absorption: OS
Pancreatic secretion: OS
Melanogenesis: OS
Glucagon signaling pathway: OS
Estrogen signaling pathway: OS
Thyroid hormone synthesis: OS
PPAR signaling pathway: OS
Antigen processing and presentation: OS
